# Neurotransmitter and Related Metabolic Profiling in the Nucleus Accumbens of Chronic Unpredictable Mild Stress-Induced Anhedonia-Like Rats

**DOI:** 10.3389/fnbeh.2022.862683

**Published:** 2022-04-29

**Authors:** Yan Li, Zhi Chen, Jianting Zhao, Heming Yu, Xiangyu Chen, Yong He, Yu Tian, Yue Wang, Chong Chen, Ke Cheng, Peng Xie

**Affiliations:** ^1^Department of Neurology, The First Affiliated Hospital of Chongqing Medical University, Chongqing, China; ^2^NHC Key Laboratory of Diagnosis and Treatment on Brain Functional Diseases, Chongqing Medical University, Chongqing, China; ^3^Department of Neurology, Xinxiang Central Hospital, The Fourth Clinical College of Xinxiang Medical College, Xinxiang, China

**Keywords:** major depressive disorder, chronic unpredictable mild stress, nucleus accumbens, neurotransmitter, 5-HT, glutamate

## Abstract

Major depressive disorder (MDD) is a serious mental disorder that affects many people. The neurotransmitter deficiency hypothesis has been the crux of much research on the treatment of depression. Anhedonia, as a core symptom, was closely associated with altered levels of 5-hydroxytryptamine (5-HT), dopamine (DA), and diverse types of glutamate (Glu) receptors in the nucleus accumbens (NAc). However, there were no reports showing how Glu changed in the NAc, and there were other unreported molecules involved in modulating stress-induced anhedonia. Thus, we investigated changes in neurotransmitters and their related metabolites in GABAergic, serotonergic and catecholaminergic pathways in the NAc of a rat model of chronic unpredictable mild stress- (CUMS-) induced anhedonia-like behavior. Then, liquid chromatography-tandem mass spectrometry (LC-MS/MS) was employed to detect target neurotransmitters and related metabolites in the NAc. Finally, the Western blot was used to assess the expression of key enzymes and receptors. Here, we found that the 5-HT level in anhedonia-susceptible (Sus) rats was increased while the Glu level decreased. DA did not show a significant change among CUMS rats. Correspondingly, we detected a reduction in monoamine oxidase-A (MAOA) and Glu receptor 1 levels in anhedonia-Sus rats while Glu receptor 2 (GluR2) and NMDA2B levels were increased in anhedonia-resilient (Res) rats. We also found that the levels of glutamine (Gln), kynurenic acid (Kya), histamine (HA), L-phenylalanine (L-Phe), and tyramine (Tyra) were changed after CUMS. These alterations in neurotransmitters may serve as a new insight into understanding the development of anhedonia-like behavior in depression.

## Introduction

Major depressive disorder (MDD) is a mental disease with many overlapping symptoms and diverse etiologies, and it is a massive social burden. Any condition that causes strong mental stress or severe and unpleasant emotional experiences may cause MDD (Cipriani et al., 2016). It has been reported that dysfunction of the brain reward pathway may contribute to the development of MDD (Russo and Nestler, 2013), which may be explained by the neurotransmitter deficiency hypothesis.

The nucleus accumbens (NAc) has attracted significant attention as a reward and emotional center of the brain (Zhu et al., 2017). Depression-like animals repeatedly showed stress-induced NAc hypertrophy, which can be reversed with ketamine (Abdallah et al., 2017). The NAc regulates emotional and reward-related stimulation by further integrating signals transferred by neurotransmitters from different regions of the limbic system (Arango-Lievano et al., 2014). Under repeated stress, the NAc shows some deficits in signaling transfer, which facilitates depression-related behaviors (Floresco, 2015; Francis and Lobo, 2017). The NAc was involved in complex interactions with the neurotransmitter signaling system. Antidepressant drugs, such as serotonin and norepinephrine reuptake inhibitors, achieved fast antidepressant effects by elevating dopamine (DA) concentrations in the NAc (Li et al., 2020). Levels of 5-hydroxytryptamine (serotonin, 5-HT) and 5-hydroxyindolacetic acid (5-HIAA) were reduced in the NAc in depressive-like rats induced by anabolic androgenic steroid abuse, while DA and its metabolites were not changed (Tucci et al., 2012). In a prenatal restraint stress rat study, male PRS rats showed upregulated DA levels and downregulated 5-HT levels in the NAc. Female PRS rats showed lower DA and 5-HT levels in the NAc (Reynaert et al., 2016). Intraperitoneal administration of the putative δ1-receptor agonist in rats induced antidepressant-like behavior accompanied by increased release of DA and L-glutamate (Glu) but decreased γ-aminobutyric acid (GABA) in the NAc (Tanahashi et al., 2012). These studies showed that neurotransmitter alterations in the NAc were closely connected to depressive-like behavior.

The chronic unpredictable mild stress (CUMS) model is a well-known paradigm to generate anxiety- and anhedonia-type behavior in rats and mice (Kompagne et al., 2008). Under CUMS exposure, depressive-like rats exhibited decreased contents of norepinephrine, 5−HT, and DA levels in the NAc (Shen et al., 2019), and the administration of L-theanine can reverse this reduction in neurotransmitters. In CUMS-induced depressive-like mice, the expression of GABA-associated mRNAs and proteins decreased (Ma et al., 2019a) while DA and its metabolites increased (Lu et al., 2019). Another high-throughput RNA sequencing research into CUMS mice showed that differentially expressed mRNAs in the NAc were significantly enriched in GABAergic synapses, dopaminergic synapses, neurotransmitter synthesis, etc. (Ma et al., 2019b). This evidence indicated that CUMS-induced depressive-like behavior had a deep relation with chaotic neurotransmitters in the NAc. Depressive-like criteria in their study were based on both behavioral despair and anhedonia. Nevertheless, in their study, the forced swimming test (FST) was one of the evaluation criteria for behavioral despair. FST remains a controversial behavior in evaluating depressive-like in rodents (Molendijk and de Kloet, 2021). Conversely, the criteria for anhedonia-the sucrose preference test (SPT) is not controversial.

There is a large consensus that anhedonia is one of the core symptoms of MDD. Anhedonia reflects deficits in the processing of reward information (Höflich et al., 2019). A neuroimaging study (Liu et al., 2021) found that the neural correlates of anhedonia were modulated by depression and that the modulatory effect was regionally dependent on the NAc. A previous study reported that higher levels of trait anhedonia were associated with reduced activity in the NAc (Keller et al., 2013; Wang et al., 2021). Meanwhile, anhedonia was accompanied by changes in neurotransmitter levels in the NAc, such as DA, 5-HT, and norepinephrine (Wang et al., 2021). It was reported that Glu receptors 1/2 (GluR1/2) (Todtenkopf et al., 2006) and NMDA receptor 2B (NMDAR2B) (Jiang et al., 2013) were involved in modulating anhedonia in rodents. However, how the glutamine (Gln) level changes were not reported in the NAc of patients with anhedonia or animals with anhedonia-like behavior. Moreover, there may exist other unrevealed molecules involved in anhedonia. Given the evidence, we hypothesized that Glu and other neurotransmitters co-regulate chronic stress-induced anhedonia. Therefore, CUMS was used to induce anhedonia-like behavior in rats. Liquid chromatography-tandem mass spectrometry (LC-MS/MS) was applied to detect the concentrations of 24 neurotransmitters and their related metabolites involved in serotoninergic, GABAergic, and catecholaminergic pathways in the NAc. Some of the key enzymes and transporters among these molecules were validated by Western blots. We aimed to explore the potential neurotransmitter alternation associated with anhedonia among CUMS rats.

## Materials and Methods

### Animals

Sprague-Dawley rats (male, 6 weeks old, body weight 200–250 g) were obtained from the laboratory animal center of Chongqing Medical University (China). All rats were housed under standard laboratory conditions (23°C ± 1°C, 45% ± 15% relative humidity, and a 12/12-h day/night cycle) with free access to a standard rat diet and tap water during the experiment. Our animal study was approved by the Ethics Committee of Army Medical University (China) and Chongqing Medical University (2011002). All experiments were performed in accordance with the National Institutes of Health Guide for the Care and Use of Laboratory Animals.

### Sucrose Preference Baseline and Body Weight Measurement

Rats were trained to drink a 2% sucrose solution for 48 h. After training with sucrose, baseline measurements were performed. Sucrose preference (SP) was calculated with the following formula: SP = (sucrose intake/total intake) × 100%. This measurement was repeated five times (each time interval of 1 day) to obtain an average SP and to exclude abnormal rats (Liu et al., 2018) (rats whose average SP < 80% or those that only drank sucrose). The initial body weight measurement was taken between 9 and 10 am before the CUMS procedure.

### Chronic Unpredictable Mild Stress Procedure

**FIGURE 1:**
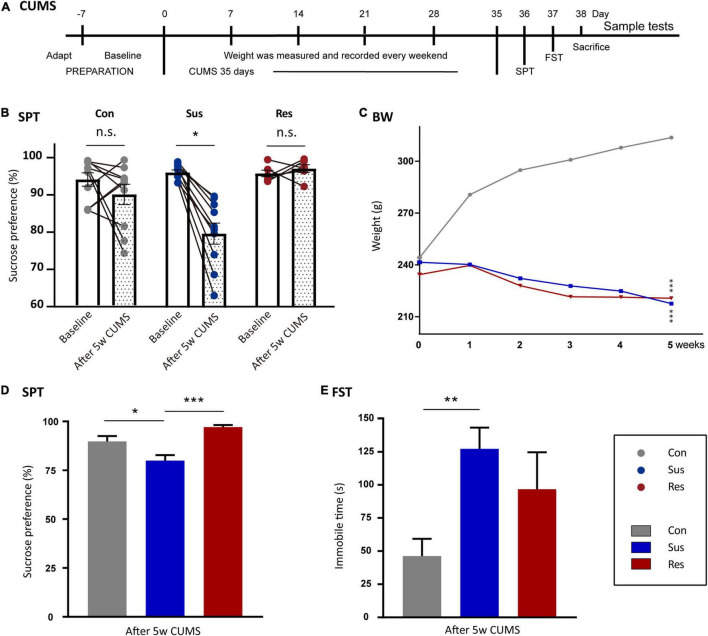
Chronic unpredictable mild stress-(CUMS-) induced depressive-like behaviors and resilience in rats. **(A)** The timeline of the CUMS model. **(B)** Sucrose preference (SP) changed before and after CUMS in the control (Ctrl, *n* = 10), susceptible (Sus, *n* = 10), and resilient (Res, *n* = 6) groups. **(C)** Body weight line chart of the Sus, Res, and Ctrl groups. “*”meant a significant difference compared with Ctrl in the body weight gain. **(D)** SP values after CUMS. **(E)** Immobility times in the three groups in forced swimming test (FST). Values are presented as means ± standard error of the mean (SEM). *p < 0.05, **p < 0.01, ***p < 0.001. The one-way repeated-measures analysis of variance (ANOVA) was used for a comparison of the body weight gain. Paired *t*-test was used to compare SP before and after CUMS. The one-way ANOVA was used to compare the three groups.

In this study, eight rats were excluded from the CUMS procedure due to abnormal SP (35 rats in total). The remaining 27 rats were randomly divided into control (Ctrl, *n* = 10) and stress (CUMS, *n* = 17) groups according to initial body weight and baseline SP. There were no significant differences between Ctrl and CUMS rats in body weight or SP. To avoid acoustic and olfactory interference during CUMS exposure, Ctrl rats were housed in a quiet and undisturbed environment. CUMS rats were subjected to unpredictable mild stress for 5 weeks (Tian et al., 2020; Figure 1A). Stressors consisted of long- (>12 h) and short-term stressors (<8 h). Long-term stressors included food and water deprivation (24 h), crowded cage (two rats per cage, 24 h), light during the dark cycle (12 h), soiled cage (24 h), tilting cage (12 h, 45°), and stroboscopic illumination (60 flashes per min, 12 h). Short-term stressors included a cold environment (4°C, 15 min), swimming (23°C, 5 min), shake cage (horizontal, speed 160, 10 min), tail pinching (1–2 min), and foot shock (2.5 mA, 10-s duration per stimulation with the same interval time, repeated four times). More than two types of stressors (the combination of at least one of the long- and short-term stressors, respectively) were used randomly in the stress group, making it impossible for rats to predict the stimulus (Table 1).

**TABLE 1:** Stressors in the chronic mild stress model procedure.

Stressors	Mon	Tue	Wed	Thu	Fri	Sat	Sun
Continuous light	√				√		
Crowded condition	√				√		
Food and Water deprivation		√					
Soiled cage				√			
Cage title						√	
Stroboscopic illumination			√				√
Cold environment				√		√	
Swimming					√		
Shake cage	√			√			√
Tail pinching		√					
Foot shock			√				

*Stressor schedule for the 1st week. In the stressed group, more than two types of stressors (combination of at least one of the long- and the short-term stressors, respectively) were used randomly. Making it impossible for the rats to predict the stimulus, the stressors varied from week to week.*

### Evaluation of the Chronic Unpredictable Mild Stress Rat Model

Body weight was measured and recorded every 7 days during the CUMS procedure. SPT was administered at the end of the 5th week using the same method as the baseline measurement. SPT was used to determine whether a rat was susceptible (Sus) or resilient (Res) to stress. For CUMS rats, rats in which SP decreased by more than 5% on day 35 were classified into the anhedonia-Sus group (Tian et al., 2020), while the remaining rats were classified into the anhedonia-Res group. FST was performed in a cylinder (50 cm height × 25 cm diameter) filled with water (up to 30 cm, at 23–25°C). During the training session, all rats were habituated to the water temperature and apparatus for 15 min. Then, 24 h later, the test was conducted for a duration of 6 min. The behaviors of each rat were auto-recorded during the test and used to identify normal activities.

### Sample Collection and LC-MS/MS Measurement

Rats were subjected to cardiac perfusion and decapitated under anesthesia (pentobarbital sodium, 50 mg/kg). Brain tissues were collected according to *The Rat Brain in Stereotaxic Coordinates (6th Edition)*. The NAc tissues were collected with pointed tweezers (it was necessary to exclude the Aca (anterior commissure and anterior part) tissue surrounded by the NAc). Then, the NAc tissues were quickly frozen in liquid nitrogen. The LC-MS/MS analysis was conducted as previously described (Wang et al., 2016).

### Western Blot

The NAc tissues from rats were lysed with RIPA buffer containing a protease and phosphatase inhibitor. The tissues were then sonicated on ice and centrifuged (12,000 × g, 4°C for 10 min). Next, in importance, equal amounts of protein samples were measured using the bicinchoninic acid (BCA) assay. Subsequently, protein samples were successively separated by 10% sodium dodecyl sulfate–polyacrylamide gel electrophoresis, transferred onto polyvinylidene fluoride membranes (Millipore, Billerica, MA, United States), and blocked with a 5% non-fat milk solution (room temperature for 2 h). Next, the membranes were incubated with primary antibodies (Supplementary Table 1) overnight at 4°C. Then, Tris-buffered saline with 0.05% Tween-20 (TBST) was used to wash the membranes, which were then incubated with respective secondary antibodies (Bio-Rad, Hercules, California) at room temperature for 2 h. Eventually, the visible signals of the protein bands, acquired *via* enhanced chemiluminescence, were quantified using the Quantity One software.

### Statistical Analyses

All results are presented as means ± standard error of the mean (SEM). Statistical analyses of all data were conducted with SPSS 25.0 software for Windows (SPSS, Inc., Chicago, IL, United States). The normality of the data was analyzed using the Shapiro–Wilk test. If it was a normal distribution, differences among the three groups were assessed by a one-way analysis of variance (ANOVA) throughout the study, and *post-hoc* tests between all groups were performed using the least significant difference (LSD) test. Otherwise, the Kruskal–Wallis test was used. Body weight was analyzed using a one-way repeated measures ANOVA with group as an independent factor and time as a repeated measure, and the value of *p* was calculated from the LSD *post-hoc* test. Paired *t*-test was used to compare SP before and after CUMS. The significance level for all tests was set at *p* < 0.05. GraphPad Prism 8.0 was used to visualize the results of statistical analyses.

## Results

### Chronic Unpredictable Mild Stress Successfully Induced Anhedonia-Like Behaviors in Rats

To evaluate anhedonia-like behaviors, related behavioral experiments were performed on all rats. In SPT, 10 rats in the CUMS group showed more than 5% reduction in SP (Tian et al., 2021) and were defined as anhedonia-Sus (Figure 1B), while the six rats in the CUMS group were designated as anhedonia-Res. After 5 weeks of CUMS, the SP values of Sus rats decreased significantly compared with baseline (*p* = 0.041, Figure 1B), while there were no significances in both the Ctrl and Res group before and after CUMS (Figure 1B). Body weight was recorded in the line graph from 0 to 5th week (Figure 1C). Body weight gains of Sus (compared with Ctrl, *p* = 0.000) and Res (compared with Ctrl, *p* = 0.000) rats were significantly decreased compared with Ctrl rats. No differences in the body weight gain were observed between Sus and Res rats. Significant differences in SP were observed in Ctrl and Sus rats (*p* = 0.010) as well as in Res and Sus rats (*p* = 0.001) [*F*_(2,23)_ = 9.456; Figure 1D]. In FST, Sus rats showed increased immobility time (*p* = 0.002) compared with Ctrl rats [*F*_(2,23)_ = 6.208; Figure 1E], while there was no difference between Sus and Res. One rat that died during the CUMS procedure was removed from further analysis.

### Differential Abundances of Neurotransmitter in the Nucleus Accumbens in Three Pathways

**FIGURE 2:**
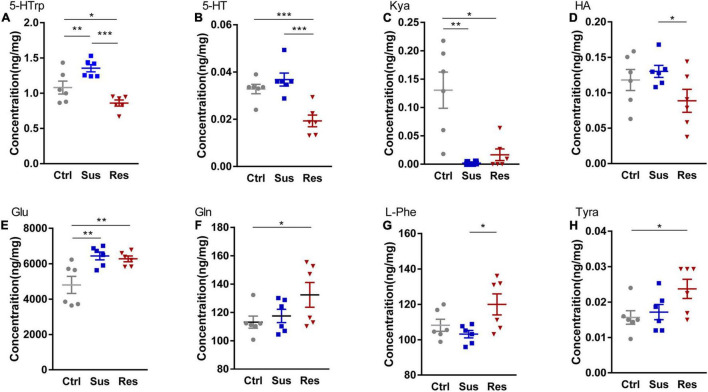
Changes in metabolites of three pathways in the nucleus accumbens (NAc). Levels of metabolites were determined by liquid chromatography-tandem mass spectrometry (LC-MS/MS), including **(A)** 5-hydroxytryptophan (5-HTrp), **(B)** 5-hydroxytryptamine (5-HT), **(C)** kynurenic acid (Kya), **(D)** histamine (HA), **(E)** glutamate (Glu), **(F)** glutamine (Gln), **(G)** L-phenylalanine (L-Phe), and **(H)** tyramine (Tyra). *p < 0.05, **p < 0.01, ***p < 0.001. One-way ANOVA and the Kruskal–Wallis test were used to compare the three groups.

To uncover alterations in neurotransmitters and related metabolites in three metabolic pathways, the levels of 24 molecules in three groups were measured (Supplementary Table 2). Eight molecules showed significant differences among these neurotransmitters. In the serotonergic pathway, 5-hydroxytryptophan (5-HTrp), was significantly increased in the Sus group (*p* = 0.010) and decreased in the Res group (*p* < 0.031) [*F*_(2,15)_ = 14.224; Figure 2A]. Additionally, 5-HT was significantly reduced in the Res group compared with the Ctrl (*p* = 0.001) and Sus (*p* = 0.000) groups [*F*_(2,15)_ = 14.285; Figure 2B]. Kynurenic acid (Kya) was significantly decreased in both the Sus (*p* = 0.07) and Res (*p* = 0.05) groups compared with Ctrl [*N*_(2,15)_ = 10.347; Figure 2C]. In addition, histamine (HA) was significantly decreased in the Res group compared with the Sus group (*p* = 0.048) [*F*_(2,15)_ = 2.458; Figure 2D]. In the GABAergic pathway, Glu was significantly increased in both the Sus (*p* = 0.003) and Res (*p* = 0.006) groups [*F*_(2,15)_ = 7.872; Figure 2E]. Gln showed a tendency toward upregulation in the Res group compared with the Ctrl (*p* = 0.045) [*F*_(2,15)_ = 2.63; Figure 2F]. In the catecholaminergic pathway, only two neurotransmitters were significantly different. L-phenylalanine (L-Phe) was increased in the Res group compared with the Sus group (*p* = 0.012) [*F*_(2,15)_ = 4.358; Figure 2G], and Tyramine (Tyra) was also increased in the Res group compared with Ctrl rats (*p* = 0.024) [*F*_(2,15)_ = 3.541; Figure 2H]. There were no significant differences in the other neurotransmitters among the three groups.

### Altered Protein Expression in the Nucleus Accumbens

**FIGURE 3:**
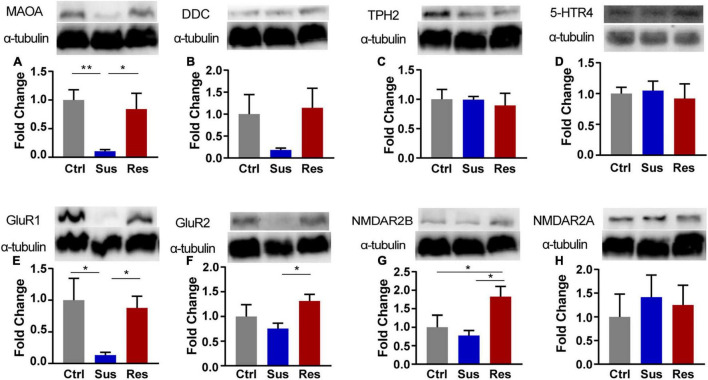
The Western blot analysis of protein expression levels of key enzymes in the monoaminergic pathway in the NAc of rats after CUMS. **(A)** monoamine oxidase-A (MAOA), **(B)** dopamine decarboxylase (DDC), **(C)** tryptophan hydroxylase 2 (TPH2), **(D)** 5-HT receptor 4 (5-HTR4), **(E)** Glu receptor 1 (GluR1), **(F)** GluR2, **(G)** NMDA receptor 2B (NMDAR2B), and **(H)** NMDAR2A. Protein expression levels were examined by the Western blot (*n* = 4 rats per group). α-Tubulin was used as a loading Ctrl. DDC and NMDAR2B, TPH, and NMDAR2A used the same loading Ctrl. *p < 0.05, **p < 0.01, *** *p* < 0.001. One-way ANOVA and the Kruskal-Wallis test were used to compare the three groups.

In response to changes in GABAergic and serotoninergic neurotransmitters, the key protein expression levels in the NAc were validated by the Western blot analysis. In the serotoninergic pathway, levels of dopamine decarboxylase (DDC), monoamine oxidase-A (MAOA), tryptophan hydroxylase 2 (TPH2), and 5-HT receptor 4 (5-HTR4) were tested. MAOA protein expression was downregulated in Sus rats (*p* = 0.009, compared with Ctrl; *p* = 0.024, compared with Res) [*F*_(2,9)_ = 6.206; Figure 3A], while there was no significant difference in the levels of DDC, 5-HTR4, or TPH2 among the three groups (Figures 3B–D). In the GABAergic pathway, GluR1/2 and NMDAR2A/2B levels were tested. Protein expression of GluR1 was significantly decreased in Sus rats among the three groups (*p* = 0.024, compared with Ctrl; *p* = 0.045, compared with Res) [*F*_(2,9)_ = 4.275; Figure 3E]. Protein expression levels of GluR2 (*p* = 0.044) [*F*_(2,9)_ = 2.746; Figure 3F] and NMDAR2B (*p* = 0.018, compared with Sus) [*F*_(2,9)_ = 4.635; Figure 3G] were increased in Res rats compared with Sus rats. No changes in protein expression levels of NMDAR2A were found among the three groups (Figure 3H).

## Discussion

In the 5th week of the CUMS procedure, reduced SP showed significant differences in Sus rats compared with Ctrl and Res rats. This result indicated that anhedonia-like behavior had been successfully induced by CUMS. Although FST was performed in our study, these rats did not exhibit depressive-like behavior. Recently, FST has been recognized as a controversial behavior for depression; the immobility response in FST may not be a rodent model of depression (Molendijk and de Kloet, 2021). According to the article by E. Ronald de Kloet (de Kloet et al., 2019; Molendijk and de Kloet, 2019), the immobility response reflects preferred memory and adaptability to behavioral despair; these are anthropomorphic interpretations. It is challenging to model psychiatric illnesses as we do not have accepted and clear etiologies. Therefore, we paid attention to the anhedonia-like and anti-anhedonia rats induced by CUMS in a consequent exploration of neurotransmitters. In the NAc of CUMS rats, eight neurotransmitters displayed significant changes among the three groups in the serotonergic, catecholaminergic, and GABAergic pathway by LC-MS/MS. The altered neurotransmitters included 5-HTrp, 5-HT, Kya, HA, Glu, Gln, L-phe, and Tyra, which showed similar or different changes among these rats.

As mentioned earlier, the NAc plays a pivotal role in anhedonia modulated by the reward pathway. Signaling in the NAc is intricate. It receives inputs from the prefrontal cortex, ventral tegmental area (VTA), amygdala, and hippocampus *via* neurotransmitters like DA, Glu, GABA, NE, and 5-HT (Shirayama and Chaki, 2006). A previous study reported that peripherally administered tumor necrosis factor-α (TNF-α) induced anhedonia-like behavior in mice, and then observed an increase in extracellular 5-HIAA and homovanillic acid (HVA) levels in the NAc without changing 5-HT and DA. For this change in 5-HT, the explanation was that TNF-α might have enhanced serotonin transporter (SERT) function and activity, leading to enhanced 5-HIAA levels in the NAc (van Heesch et al., 2013). However, we found that the concentration of 5-HT and 5-HTrp (a substrate that can generate 5-HT by decarboxylation) was upregulated in anhedonia-like rats but decreased in anti-anhedonia rats, while 5-HIAA and HVA levels did not change. We also detected the expression of MAOA (the key enzyme of the 5-HT catabolism) protein in the NAc. The protein level of MAOA was significantly decreased in anhedonia-like rats, indicating that 5-HT might not be catabolized and therefore increased in the NAc. This difference in the 5-HT level is probably due to anhedonia from a different source (CUMS-induced rats in our study). Thus, a higher 5-HT level in the NAc may be one of the factors modulating stress-induced anhedonia-like behavior.

Two types of neurons are involved in DA in the NAc: medium spiny neuron-(MSN D1 and MSN-D2). In DA transmission from the VTA to the NAc, the inhibition of D1-MSNs increased vulnerability to anhedonia-like symptoms, such as decreased SP and social interaction (Coccurello, 2019). However, a recent study associated with anhedonia certified that stress-elicited VTA GABA, but not DA, neurons activity mediated blunted reward-seeking (Lowes et al., 2021). Thus, it may be explainable in our results that DA did not show alterations in the NAc of anhedonia-like rats.

Glutamate is a major excitatory neurotransmitter in the brain. NMDA receptors are densely expressed in MSNs in the NAc (Jiang et al., 2013), where most were efferent GABAergic. The activity of these neurons in the NAc is significantly regulated by glutamatergic afferents from the amygdala and hippocampus (Shirayama and Chaki, 2006). Moreover, disturbances in glutamatergic neurotransmission and synaptic plasticity in NAc regions might indirectly lead to anhedonia and depression (Jiang et al., 2013). Long-term potentiation (LTP) at hippocampal-NAc synapses in reward driven motivational behaviors such as anhedonia involved canonical NMDA receptor-dependent mechanisms, but did not require DA signaling (Wang et al., 2010). Thus, anhedonia-like behavior in rats may need a higher level of Glu involved in these processes in the NAc. This may be the reason for the elevated Glu level and the unchanged DA level in anhedonia-like rats.

Interestingly, we also found that Glu and Gln presented different alterations in the NAc of CUMS rats, which may be related to the metabolic exchange between Glu and Gln. Glu is metabolized into Gln by glutamine synthase, and Gln is released by astrocytes in pre-projecting neurons and converted back to Glu by cytosolic glutaminase (Lener et al., 2017). In this case, we observed that Gln level decreased in Sus rats compared with Res rats while Glu levels were increased in both Sus and Res groups. In summary, only Gln was reduced in Sus. In a study of chronic social defeated stress (CSDS) rats, researchers found reduced extracellular Gln levels in males under stress exposure (Rappeneau et al., 2016). Thus, we speculated that the imbalance of interconversion between Glu and Gln might have a potential connection to susceptibility or resilience to anhedonia. This requires further validation of key enzymes and transporters in the Glu/Gln cycle.

In addition, the expression of Glu receptor levels had also changed in this study. It has been reported that elevated GluR1 in the NAc shell increases ICSS [intracranial self-stimulation, upgrade ICSS indicative of an anhedonic state (Redgrave and Dean, 1981)] thresholds, while elevated GluR2 decreased ICSS thresholds (Todtenkopf et al., 2006). GluR1 and GluR2 in the NAc shell play opposing roles in regulating motivated behavior. Here, we found that the protein of GluR1 was reduced in Sus rats and GluR2 was increased in Res rats. GluR2 may act as an effect of anti-anhedonia. In addition, our result was based on the whole NAc rather than the NAc shell, which may explain the different levels of GluR1 with their study. In another study of CSDS mice, the author found decreased NMDAR2B expression in the NAc of Sus mice (Jiang et al., 2013). They used the proteasome inhibitor MG132 to reverse the loss of NMDAR2B and then restored reduced SP and social interaction. We observed increased NMDA2B in Res rats and unchanged NMDA2A levels, consistent with their result. Glu may regulate anhedonia-like behavior through these receptors in the NAc.

Recently, Kya has been well-established as an antagonist at all Glu receptor subtypes (Xu et al., 2019), and it may have the ability to modulate extracellular Glu levels in the brain. Kya Ctrls glutamatergic and dopaminergic neurotransmission, and elevated brain levels appear to be related to psychotic symptoms and cognitive impairments (Erhardt et al., 2017). We found that Kya levels were decreased in both Sus and Res rats. It may affect the Glu level in the NAc.

## Conclusion

Although neurotransmitters in anhedonia-like rats were measured, there are some limitations in this study. The volume of the NAc is quite small, and accordingly, we could not verify all the enzymes and transport of neurotransmitters in the three pathways by the Western blot and real-time quantitative polymerase chain reaction (RT-qPCR); this remains to be investigated in future studies. Here, we showed that 5-HT and Glu changes in the NAc may be involved in stress-induced anhedonia. However, this study paid attention only to neurotransmitter changes in the NAc in anhedonia-like behavior. To determine the mechanism of these neurotransmitter alterations, more experiments are needed. In summary, this study provides a novel insight into the molecular changes in the NAc in anhedonia-like behavior of depression.

## Data Availability Statement

The raw data supporting the conclusions of this article will be made available by the authors, without undue reservation.

## Ethics Statement

The animal study was reviewed and approved by the Ethics Committee of Army Medical University (China) and Chongqing Medical University (2011002).

## Author Contributions

KC: conceptualization. YT: methodology. YW and YH: resources. YL and ZC: animal operation and writing–original draft. ZC and HY: formal analysis. YH and CC: data curation. YL and XC: visualization. YT and KC: writing–review and editing. PX and KC: supervision. All authors contributed to the article and approved the submitted version.

## Conflict of Interest

The authors declare that the research was conducted in the absence of any commercial or financial relationships that could be construed as a potential conflict of interest.

## Publisher’s Note

All claims expressed in this article are solely those of the authors and do not necessarily represent those of their affiliated organizations, or those of the publisher, the editors and the reviewers. Any product that may be evaluated in this article, or claim that may be made by its manufacturer, is not guaranteed or endorsed by the publisher.
