# Supplementary material for: Neurotransmitter and Related Metabolic Profiling in the Nucleus Accumbens of Chronic Unpredictable Mild Stress-Induced Anhedonia-Like Rats
Source: Front Behav Neurosci. 2022 Apr 29;16:862683. doi: 10.3389/fnbeh.2022.862683 (PMC9100667; doi:10.3389/fnbeh.2022.862683)
Supplement: Supplementary file 1 [file Data_Sheet_1.docx]

**Table S1 Antibody used in western blot.**

| Antibody | Dilution | Catalogue numbers | Source |
| --- | --- | --- | --- |
| DDC | 1:1000 | 13561S | Cell Signaling Technology |
| MAOA | 1:500 | ab200928 | Abcam |
| TPH2 | 1:1000 | 51124S | Cell Signaling Technology |
| GluR1 | 1:1000 | ab109450 | Abcam |
| GluR2 | 1:300 | 32-0300 | Invitrogen |
| NMDAR2A | 1:1000 | ab124913 | Abcam |
| NMDAR2B | 1:1000 | ab183942 | Abcam |
| 5-HTR4 | 1:500 | 13690S | Cell Signaling Technology |
| α-tubulin | 1:5000 | ab176560 | Abcam |

**Table S2 Concentration (ng/g) of NAc NTs**

|  |  | Con (n=6) | Sus (n=6) | Res (n=6) |
| --- | --- | --- | --- | --- |
| Tryptophanic  pathway | Trp | 12.4466±0.7714 | 14.4858±0.7717 | 12.9125±0.4476 |
|  | 5-HTrp | 1.0805±0.0916 | 1.3541±0.0508^**,###^ | 0.8602±0.0440^*^ |
|  | 5-HIAA | 2.7837±0.2817 | 3.4184±0.2549 | 3.6951±0.3696 |
|  | Tra | 0.0043±0.0006 | 0.0032±0.0006 | 0.0038±0.0006 |
|  | Trpo | 0.2595±0.0749 | 0.1307±0.0183 | 0.2056±0.455 |
|  | Kyn | 0.3005±0.0917 | 0.0773±0.0264 | 0.0156±0.0051 |
|  | Kya | 0.1305±0.0319 | 0.0033±0.0008^**^ | 0.0333±0.0113^*^ |
|  | 3-HAphe | 1.6620±0.1447 | 1.8146±0.1685 | 1.7261±0.1995 |
|  | 5-HT | 0.0328±0.002 | 0.0368±0.0028^***,###^ | 0.0193±0.0025 |
|  | Ind-3-C | 0.0136±0.0015 | 0.0172±0.0037 | 0.0231±0.0016 |
|  | HVA | 7.7953±0.5914 | 7.8035±0.5207 | 8.8266±0.6935 |
|  | Ind-3-L | 0.1441±0.0068 | 0.0698±0.0189 | 0.1294±0.0331 |
|  | IDP | 7.6588±1.0659 | 12.7625±2.0082 | 9.8366±1.8489 |
|  | HA | 0.1179±0.0148 | 0.1302±0.0086^#^ | 0.0886±0.0163 |
| GABAergic  pathway | a-KG | 2.2853±0.7224 | 0.4816±0.1073 | 1.5138±0.4101 |
|  | Glu | 4800.6881±483.6338 | 6441.1214±226.1849^**^ | 6275.2009±160.3313^**^ |
|  | GSH | 5.0311±1.77 | 9.2134±0.8802 | 6.1602±1.1347 |
|  | Gln | 113.1815±4.2225 | 117.5238±4.739 | 132.4299±8.7308^*^ |
|  | GABA | 505.563±24.1736 | 487.0358±19.7437 | 582.394±47.7005 |
| Catecholamin- ergic pathway | Phe | 108.189±3.3892 | 103.1813±2.1191^#^ | 119.9775±5.9335 |
|  | Pht | 0.0049±0.0007 | 0.0052±0.0005 | 0.0056±0.001 |
|  | Tyr | 57.6572±1.8051 | 57.4313±2.8021 | 61.1837±2.6999 |
|  | Trya | 0.0157±0.0019 | 0.0172±0.0022 | 0.0237±0.0027^*^ |
|  | L-DOPA | 2.1703±0.4368 | 2.3218±0.4521 | 2.0249±0.4165 |

^*^*P* < 0.05, ^**^*P* < 0.01, ^***^*P* < 0.001(compared with Ctrl rats); ^#^*P* < 0.05, ^##^*P* < 0.01, ^###^*P* < 0.001(compared with Res rats);

Abbreviations Trp: tryptophan; 5-HTrp: 5-hydroxytryptophan; Trpo: tryptophole; 5-HT: 5-hydroxytryptamine; 5-HIAA: 5-hydroxyindoleacetic acid; Kyn: kynurenine; Kya: kynurenic acid; Ind-3-C: indole-3-carboxaldehyde; HVA: Homovanillic acid; Ind-3-L: Indolelactic acid; IDP: indole pyruvate; HA: histamine; GABA: gamma-aminobutyric acid; α-KG: alpha-ketoglutaric acid; Glu: glutamate; Gln: glutamine; GSH: glutathione; Phe: L-phenylalanine; Pht: phenylethylamine; Tyr: L-tyrosine; Tyra: tyramine; L-DOPA: L-dopamine.
